# The impact of the COVID-19 Pandemic on breast cancer incidence and stage: Results of a retrospective study

**DOI:** 10.5339/qmj.2025.85

**Published:** 2025-09-15

**Authors:** Agnieszka Żyromska, Iga Racka, Karolina Majewska

**Affiliations:** 1 Department of Radiotherapy, Oncology Center of Professor Franciszek Łukaszczyk in Bydgoszcz, Bydgoszcz, Poland; 2 Department of Medical Physics, Oncology Center of Professor Franciszek Łukaszczyk in Bydgoszcz, Bydgoszcz, Poland *Email: agnieszkazyromska7@gmail.com

**Keywords:** Breast cancer, preventive examination, mammography, age groups, quarantine restrictions, radiation therapy, delayed diagnosis

## Abstract

**Objective:**

The study was conducted to determine the impact of the COVID-19 pandemic on the incidence and stage of breast cancer in different age groups.

**Methods:**

This retrospective study analyzed changes in the epidemiological and clinical structure of the cohort of patients treated in the radiotherapy department of the Bydgoszcz Oncology Centre in Poland from 2016 to 2023.

**Results:**

The results of the study showed that in the group of patients under 39 years of age, an increase in the incidence rate was recorded in 2020, and a decrease in 2021 to 2023. A decrease in the incidence rate was also observed in the group of patients aged 40 to 49 years in the period 2021 to 2022 and in the groups 50 to 59, 60 to 69, and >80, from 2020 to 2023. An increase in the incidence rate was recorded in the group of patients aged 70 to 79 years in 2020, 2022, and 2023, and a decrease was observed in 2021. In the group of patients under 40 years of age, the impact of the pandemic on reducing the number of cases of stage I cancer was recorded in 2020, 2022, 2023, stage IIa—in 2020, stage IIIa—from 2020 to 2023, stage IIIb—in 2020. Instead, in 2020, the number of cases of stage IIb and stage IIIc breast cancer increased, and the rate of stage IV cancer detection increased significantly, which remained high throughout the pandemic. In the group of patients over 40 years of age, a decrease in the detection rate of stage I cancer was observed from 2020 to 2023, and stage IIa in 2020. An increase in stage IIIa cancer detection was recorded in 2020, stage IIIb in 2021 to 2023, and a significant increase in stage IV detection from 2020 to 2023.

**Conclusion:**

The results demonstrated that the COVID-19 pandemic has become one of the important factors in the delayed diagnosis of breast cancer.

## 1. INTRODUCTION

Breast cancer mortality has reduced dramatically in recent decades due to oncology discoveries like efficient treatments and preventive diagnostics.^1^ Regular mammography exams have detected tumors early, improving patient outcomes. However, the coronavirus disease caused by severe acute respiratory syndrome-related coronavirus 2 (SARS-CoV-2) has caused a global health crisis, triggered by quarantine, self-isolation, and redirection of a significant part of the medical sector resources to combat COVID-19. These restrictions have led to a significant reduction in the number of preventive examinations of women, including mammography, which has jeopardized the possibility of early detection of cancer.^2–4^

Eijkelboom et al^5^ examined how the COVID-19 pandemic affected breast cancer incidence and stage in the Netherlands and Norway. The researchers matched three waves of COVID-19 incidence data to 3 years before the pandemic. The research suggested a link between morbidity changes and the suspension of screening programs, pandemic-related medical avoidance, and other unknown reasons. Nguyen et al^6^ identified the impact of barriers to access to diagnostic services for patients from racial/ethnic minority groups and patients with low socioeconomic status.

Another significant aspect to investigate is the lack of data on the reduction in preventive diagnostic measures for breast cancer detection following the implementation of quarantine restrictions. Cairns *et al* ^7^ analyzed the impact of the COVID-19 pandemic on women’s preventive screening and surgical treatment of breast cancer. Their findings showed that from 2019 to 2020, the number of preventive mammograms decreased by 44% and diagnostic mammograms by 21%, with no statistically significant difference in the number of surgeries performed or clinical stages of cancer. Negrao *et al* ^8^ demonstrated that in March to October 2020 (COVID period), the number of breast cancer diagnoses decreased by 48.7% relative to the same period in 2019. However, breast cancer cases diagnosed during the pandemic tended to present with more advanced or severe characteristics, such as higher tumor grades or later stages at diagnosis. Voigtländer *et al* ^9^ observed a 4.9% reduction in the number of registered breast cancer diagnoses in Germany. Incident malignant neoplasms dropped from 42,857 cases in the pre-pandemic period to 39,980 cases in the pandemic period (−6.7%; 95% CI, −8.7% to −4.7%). Following Guével *et al*,^10^ the number of diagnosed cases of breast cancer in 2020 decreased by 18% compared to 2019. A high level of decline in breast cancer detection due to the suspension of the regular breast cancer screening program was identified in a study by Eijkelboom et al.^11^ In 2020, it was 67%.

Due to differences in diagnostic and treatment resources, healthcare quality, and national health system organization during the COVID-19 pandemic, breast cancer case reduction rates vary widely across countries. Due to resource limitations, a small healthcare workforce, and poor digital infrastructure, certain countries’ routine cancer screening and treatment routes were disrupted by the pandemic.

The incidence rate and stages of identification of breast cancer must be studied using clinical data from one medical institution before and after the COVID-19 pandemic to objectively analyze its effects. The study examines how the COVID-19 pandemic has affected breast cancer incidence and stage by identifying changes in the epidemiological and clinical structure of a Polish cancer center’s patient cohort before and during the pandemic and by analyzing cancer stage distribution in groups of patients under and over 40 years, who have different disease prognoses. This study examines how pandemic disruptions have affected breast cancer incidence and diagnosis in Poland. Understanding these dynamics in Poland may help the worldwide community comprehend the pandemic’s impact on cancer care by providing insights into similar healthcare systems.

## 2. MATERIALS AND METHODS

The study employed a retrospective cohort sampling technique. This type of sampling involves looking back at historical data to identify groups of individuals who share common characteristics or experiences within a specified time period. In this case, the researchers reviewed medical records of patients treated at the Bydgoszcz Oncology Centre from 2016 to 2023.^12^ The Bydgoszcz Oncology Centre is one of Poland’s leading oncological institutions with a modern infrastructure and a large patient flow, which ensures the reliability of the data collected. At the same time, the survey results should be interpreted with caution, as they may not fully reflect the situation in less affluent regions of the country. This study aligned with the ethical principles of research, including anonymity, confidentiality, and beneficence. Ethical approval of the study was obtained from the Health Research Ethics Commission of the Bydgoszcz Oncology Centre with No. RA-169, September 3, 2024.

**Figure 1:**
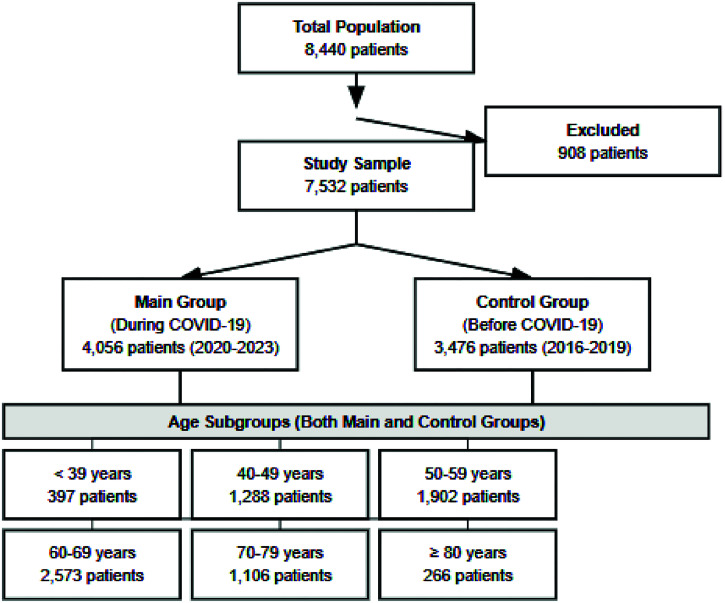
Study design.

The study included patients treated in the radiotherapy department of the cancer center from 2016 to 2023. The inclusion criteria were: (1) confirmed diagnosis of breast cancer; (2) receipt of radiotherapy as part of treatment. The exclusion criterion was the absence of data on the stage of tumors in the patient’s medical records. Thus, 908 people were excluded from the total number of patients (8,440), making a total sample of 7,532. Patients were divided into two groups: the experimental group included 4,056 patients treated in the radiotherapy department from 2020 to 2023, and the control group included 3,476 patients treated from 2016 to 2019. The allocation was performed under the official dates of the beginning and end of the COVID-19 pandemic in Poland—March 20, 2020, and July 1, 2023, respectively.^13,14^ Both groups were divided into age subgroups of patients, in which changes in their epidemiological and clinical structure before and during the pandemic were analyzed. The six subgroups included patients aged under 39 years: 397 people, 1,288 people aged 40 to 49 years, 1,902 people aged 50 to 59 years, 2,573 people aged 60 to 69 years, 1,106 people aged 70 to 79 years, and 266 people aged over 80 years (Figure 1).

Given the characteristic differences in the prognosis of the course of the disease, the distribution of cancer stages in two age groups of patients—before and after forty years—was also analyzed. The group under 40 years included 473 patients, and the group over 40 years—7,059. The incidence formula is determined as the ratio of cases within each age or stage subgroup to the total sample in the corresponding group:


(1) $$\text{Incidentrate}\;\left(\%\right)=\left(\frac{\text{Number}\;\text{of}\;\text{cases}\;\text{in}\;\text{subgroup}}{\text{Total}\;\text{number}\;\text{of}\;\text{patients}\;\text{in}\;\text{the}\;\text{group}}\right)\times 100$$

Data on demographic characteristics and tumor stage according to the 8th edition of the American Joint Committee on Cancer (AJCC)^15^ were collected by reviewing electronic medical records of patients. They were compared between the experimental and control groups, as well as between age subgroups in each of them. To identify changes in cancer incidence and stage of detection depending on the time of the pandemic, a graph showing the development of the process over time was used. Statistical analysis of clinical data was performed using PSPP software (GNU PSPP v.2.0.0). A comparison of breast cancer incidence rates and stages between the pre-and post-pandemic groups and between age groups was performed using the chi-square test. The level of statistical significance was set at *p* ≤ 0.05.

## 3. RESULTS

The primary analysis of the study groups showed that the incidence of breast cancer in the intervention group was 7.7% higher than in the control group (Table 1).

In age subgroups, the percentage distribution of this indicator was heterogeneous, as among patients under 39 years of age, aged 39 to 40 years, and 70 to 79 years, the incidence before the pandemic was lower by 0.98%, 3.28%, and 3.1%, respectively. In contrast, in the age subgroups 50 to 59, 60 to 69, and ≥80 years, the incidence was higher during the COVID-19 pandemic by 3.06%, 3.77%, and 0.44%, respectively (Table 1).

**Figure 2:**
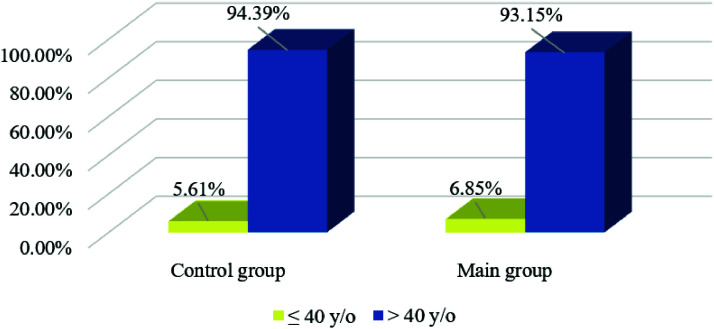
Percentage of breast cancer incidence before and during the COVID-19 pandemic in age groups under and over 40 years. Source: Compiled by the author.

The highest incidence rates were observed in the 60 to 69 and 50 to 59-year-old groups. In addition, both have seen a decline since 2020. These results indicate that the COVID-19 pandemic had a mixed impact on the incidence rate in different age groups. As different incidence dynamics were noted, it is possible to assume that age-related factors affect the development of breast cancer during the pandemic. The number of patients under 40 years increased by 1.24% from 2020 to 2023 compared to the pre-pandemic period (Figure 2).

As the COVID-19 pandemic has been complicated by global and local outbreaks, new virus strains, and pandemic response measures like quarantine, self-isolation, and vaccination, breast cancer incidence rates must be tracked over time. The 2016 to 2023 graph of breast cancer incidence revealed that from 2019 to 2020, more individuals in all age groups needed radiation. Comparing the average incidence rates from 2016 to 2019 and 2020 to 2023 is erroneous because breast cancer rates were rising before the pandemic.^16–18^ Thus, by comparing the annual increase (or decrease) in patient numbers in each age group, the pandemic’s impact on incidence rates may be objectively assessed.^19^

**Figure 3:**
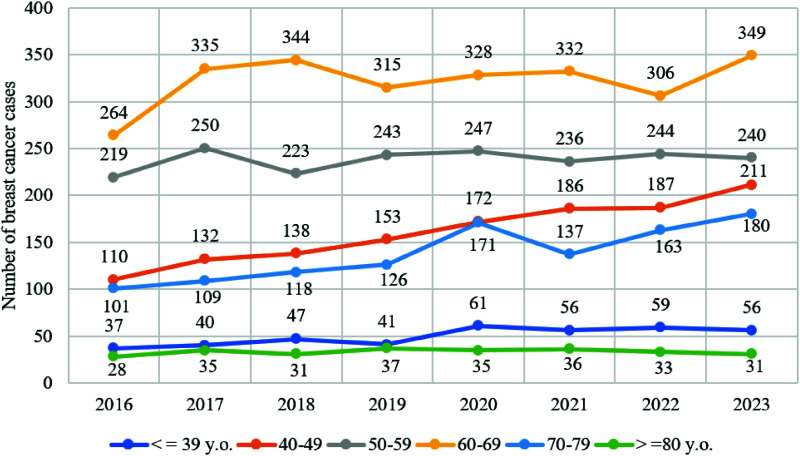
Dynamics of breast cancer incidence depending on the time of the COVID-19 pandemic development.

The biggest rise in breast cancer cases between 2019 and 2020 was 48.8% among individuals under 39. The incidence was 8.1% higher from 2016 to 2017, 17.5% higher from 2017 to 2018, and 12.8% lower from 2018 to 2019. 2021 had an 8.2% drop from 2020, followed by a 5.4% increase in 2022. Between 2022 and 2023, it fell 5.1% again. From 2020 to 2023, the average rise was 10.2% and from 2016 to 2019, 4.1%. From 2019 to 2020, the COVID-19 pandemic increased the incidence rate in patients under 39 years old (the quantitative annual increase in patients was 48.8%, compared to the average increase in the pre-pandemic period of 4.2%), but from 2021 to 2023, it decreased (a decrease of 2.6%, compared to an increase of 4.2%; Figure 3).

The number of breast cancer cases increased most linearly among patients aged 40 to 49 years. This group had a 20% peak between 2016 and 2017 and a 0.5% low between 2021 and 2022. In the four years preceding the pandemic, annual growth averaged 11.8%; during it, 8.4%. From 2020 to 2022, it fell, then rose 12.8% from 2022 to 2023. The pandemic reduced breast cancer incidence by 1.4 times. Note that 2021 to 2022 had the lowest growth (0.5%), while other periods before and after the epidemic had 11%. Thus, only from 2021 to 2022 did the pandemic reduce morbidity in people aged 40 to 49 years.

For patients aged 50 to 59 years, breast cancer incidence increased the most from 2016 to 2017 (14.2%). The reduction was 10.8% from 2017 to 2018, and the growth was 9% from 2018 to 2019. The group’s average annual incidence rate increase was 4.13% before the pandemic. This rise has slowed since 2020. It declined 4.5% and 1.6% in 2021 and 2023, respectively, from 1.6% in 2020 and 3.4% in 2022. From 2020 to 2023, this cohort had a 0.28% decline in cases, suggesting that the COVID-19 pandemic reduced the incidence rate in the 50- to 59-year age group.

The incidence rate was higher among 60 to 69-year-olds. Its evolution was non-linear. This group’s patient count increased 26.9% between 2016 and 2017. The next year, it fell to 2.7%, and in 2019, it fell 8.4%. The pandemic began with a 4.1% growth, then 1.2% in 2021, 7.8% in 2022, and 14.1% in 2023. In the control group, the average rise in patients was 7.1%, and in the experimental group, 2.9%, indicating that the COVID-19 pandemic reduced the incidence rate among patients aged 60 to 69 years.

Breast cancer rates in 70- to 79-year-olds differed greatly before and after the epidemic. This development is notable because this group’s incidence rate increased little but linearly from 2016 to 2019. From 2016 to 2017, the growth was 7.9%, from 2017 to 2018, 8.3%, from 2018 to 2019, 6.8%, and from 2019 to 2020, 36.5%. From 2020 to 2021, the incidence rate dropped 20.3%, then rose 19% and 10.4% from 2021 to 2022 and 2022 to 2023. Although the percentage increase (and decrease) in breast cancer cases from 2020 to 2023 is heterogeneous, the COVID-19 pandemic has contributed to an increase in the incidence rate among people aged 70 to 79 years in 2020, 2021 to 2022, and 2022 to 2023, and a decrease in 2021.

The impact of the coronavirus pandemic on breast cancer incidence was clearest in people over 80 years old. Despite the 25% increase in patients from 2016 to 2017, 11.4% drop from 2017 to 2018, and 19.4% increase from 2018 to 2019, the incidence rate in this group increased 11% annually. In 2020, 2022, and 2023, the incidence rate dropped 5.4%, 8.3%, and 6.1%, respectively, and rose 2.9% in 2021. Thus, the COVID-19 pandemic may have reduced the incidence rate in persons over 80 years.

**Figure 4:**
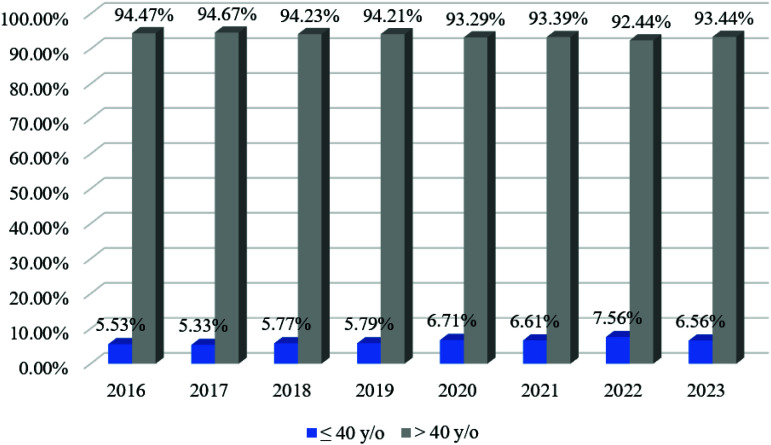
Percentage distribution of breast cancer incidence depending on the time of the COVID-19 pandemic in groups of patients under and over 40 years of age.

**Figure 5:**
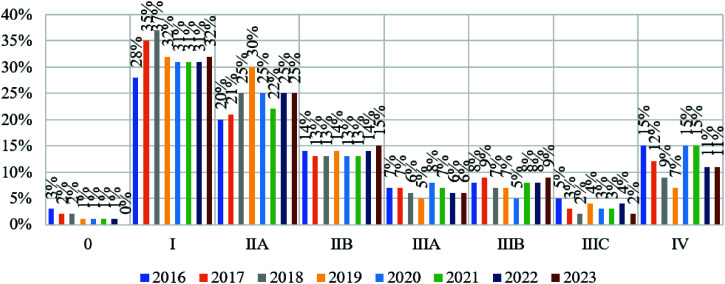
Percentage distribution by stage of breast cancer detection depending on the time of the COVID-19 pandemic development.

A downward trend in breast cancer incidence during the pandemic was noted in the group of patients over 40 years old, which is consistent with the trend in the age groups of patients aged 40 to 80 years and older. In the group of patients under 40 years, on the contrary, an increase in the incidence rate has been recorded since 2020 (Figure 4). The percentage distribution by stage of breast cancer detection before and during the pandemic is shown in Table 1. The number of cases of stage 0 decreased by 1% and stage I by 2%; the number of cases of stage IIb increased by 1%, stage IIIa by 1% and stage IV by 3%. There were no changes in the number of stages IIIb and IIIc detections. The dynamics of changes in the detection of breast cancer stages depending on the time of the pandemic are shown in Figure 5.

Stage 0 breast cancer detection declined in 2018 and remained at 1% from 2018 to 2022, then dropped to 0% in 2023. Regular breast cancer screening and early diagnosis may explain why most women with breast cancer are diagnosed at stage I. Cases peaked in 2018 (37%), then dropped 5% in 2019. In 2020, diagnoses dropped 1% from 2019 and stayed at 31% until 2022, then rose to 32% in 2023.

Stage IIa breast cancer detection increased from 2016 to 2019 and declined in 2020 from 2018. It was 25% throughout the pandemic, except for 2021, when it dropped 1%. Stage IIIb detection rates did not vary before and during the pandemic, although they increased by 1% from 2022 to 2023. From 2017 to 2019, stage IIIa breast cancer detection declined by 1%, then grew by 3% from 2019 to 2020. It fell by 1% annually from 2020 to 2022 and stayed at 6% in 2023, not reaching the pre-pandemic low of 5%.

Stage IIIb breast cancer cases dropped in 2017 and stayed at 7% in 2019. This figure dropped 2% in 2020, although it rose again to pre-pandemic levels in the years following. Both before and after the pandemic, stage IIIc breast cancer detection was unequal. From 2016 to 2018, it dropped 2% and 1%, then rose 2% in 2019. The number of cancer cases at this stage reduced by 1% at the start of the pandemic, climbed by 1% from 2021 to 2022, and decreased by 2% in 2023. Since 2016, stage IV cancer detection has decreased linearly by 3% from 2016 to 2018 and by 2% from 2018 to 2019. In 2020, it rose 8%, stayed at 15% in 2021, and fell 4% in 2022–2023. These results showed that during the pandemic, stage 0, I, and IIa breast cancer detection reduced, while IIb, IIIa, IIIb, and IV rose.

**Figure 6:**
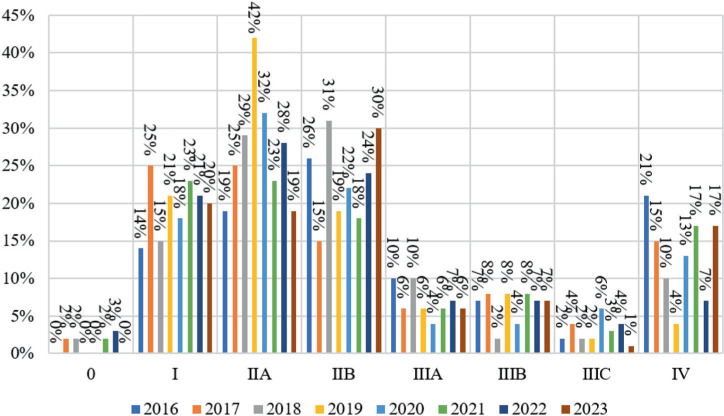
Percentage distribution by stage of breast cancer detection depending on the time of the COVID-19 pandemic development among patients under 40 years of age.

**Figure 7:**
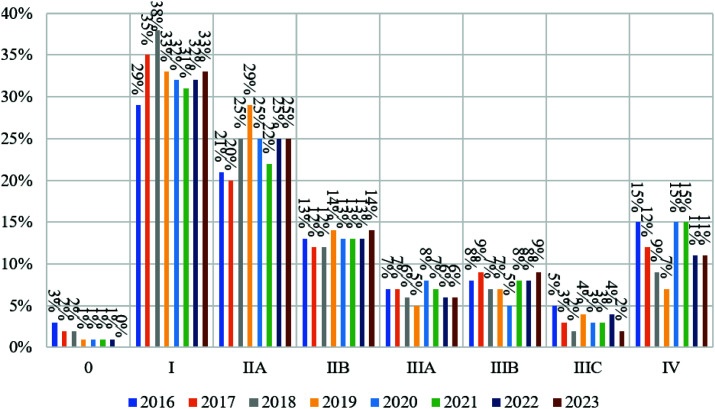
Percentage distribution by stage of breast cancer detection depending on the time of the COVID-19 pandemic among patients over 40 years of age.

As patients under 40 years and over 40 years have varying disease prognoses, it is important to analyze changes in cancer detection and pandemic dynamics in each age group (Table 2; Figures 6 and 7).

During the COVID-19 pandemic, there was an increase in the number of diagnosed breast cancer cases in this age group by 1.53%, stage I by 1.18%, stage IIb by 0.32%, stage IIIb by 1.04%, and stage IV by 1.52%. The decrease in diagnosed cancer cases was recorded for stages IIa and IIIa by 3.69% and 1.93%, respectively. Changes in the number of stage 0 breast cancer detections were not statistically significant (*p* > 0.05).

No substantial pandemic impact was seen on stage 0 breast cancer detection in this cohort. Statistically, its 2022 growth of 1% over the pre-pandemic period is satisfactory. The highest number of stage I cancer cases in this age group was in 2017. It decreased 3% from 2019 to 2020, climbed 5% in 2021, and then decreased linearly until 2023. Stage IIa breast cancer diagnoses climbed linearly until 2019, then declined by 10% in 2020, 9% in 2021, 9% in 2023 compared to 2022, and increased by 5% in 2022.

The most stage IIb cancer cases occurred in 2018 and 2023. There was no linear association between the annual proportion of instances of this stage till 2020. From 2019 to 2020, instances rose 3%, up 6% from 2022 to 2023, and down 4% from 2020 to 2021. Stage IIIa and IIIb breast cancer cases decreased by 2% and 4%, respectively, during 2019 and 2020. Notably, the number of stage IIIb cancer cases detected since 2021 returned to 2016, 2017, and 2019, which may indicate the initial impact of the pandemic, while stage IIIa cancer cases from 2021 to 2023 returned to 2017 and 2019, but not to 10%, as in 2016 and 2018. This shows that this step of cancer detection was affected throughout the pandemic.

The pandemic initially boosted stage IIIc cancer diagnoses by 4% from 2019 to 2020. Later years indicated the heterogeneity of this impact vector, but stage IIIc cancer cases in 2021-2023 dropped to pre-COVID levels. The greatest number of stage IV breast cancer cases was 21% in 2016, then fell linearly and continuously until 2019, reaching 4%. The pandemic boosted it by 9%, 4% in 2021, 10% in 2022, and 10% in 2023. This group saw the greatest increase in stage IV cancer diagnoses during the pandemic.

This data shows that breast cancer cases decreased (stages I, IIa, IIIa, and IIIb) and increased (stages IIb and IIIc) in 2020. At the start of the pandemic, stage IV cancer diagnoses increased, but they reverted to pre-pandemic levels in the following years. Thus, the pandemic decreased the number of identified stages I, IIa, IIIa, and IIIb breast cancer cases in people under 40 years and increased the number of confirmed stage IIb and IIIc cases. Stage IV cancer cases increased throughout the epidemic, causing the most damage.

The distribution of breast cancer stages in the group of patients over 40 years of age differed from the similar distribution in the group of younger patients. Stage 0 cancer was detected 1.02% more often among these patients before the pandemic than during the pandemic. The most diagnosed disease in this age group was stage I breast cancer, which decreased by 2.05% from 2020 to 2023. During the pandemic, there was also a slight decrease in the detection rates of stage IIIb (0.07%) and stage IIIc (0.52%). Between 2020 and 2023, the detection rate for stage IIa (0.39%), stage IIb (0.41%), stage IIIa (0.41%), and stage IV (2.46%) increased. The most statistically significant changes were associated with a decrease in the number of stage I cancer cases and an increase in stage IV cancer cases, which indicates the impact of the pandemic on this process.

In 2016, stage 0 breast cancer was most common in women over 40, but it then decreased until 2023. Since 2019 and 2020 showed no changes, its slow reduction suggests no pandemic impact. The detection rate for stage I breast cancer rose from 29% in 2016 to 38% in 2018. It then dropped to 32% from 2020 to 2023. Same with stage IIa cancer detection, which rose from 21% in 2016 to 29% in 2019 and 4% in 2020. The fact that from 2021 to 2023, the incidence of detection of this stage of the disease reappeared and slightly exceeded 2016—1018 numbers suggests that the pandemic only affected this group of patients at the start. From 2019 to 2020, stage IIb breast cancer detection rates dropped slightly, but not enough to affect pre-pandemic values.

From 2016 to 2019, stage IIIa cancer detection declined, then in 2020 it grew by 3%, then decreased from 2021 to 2023 to 2018. We may also argue that the pandemic had the greatest impact on stage IIIa cancer identification at the start. Stage IIIb detection declined dramatically in 2020, but from 2021 to 2023 it rebounded to 2016 to 2019 levels, with an upward trend. Thus, stage IIIb breast cancer detection shows how the pandemic affects each stage. The percentage distribution of stage IIIc cancer detection from 2016 to 2023 was similar, while the quantitative variations between groups exposed before and after 2020 were not statistically significant. Similar to younger individuals, the stage IV breast cancer detection by 2020 in people over 40 years decreased linearly from 2016 to 2019. In the first year of the pandemic, stage IV cancer cases increased 8% (9% in people under 40 years). The number of patients over 40 years remained at 2020 levels in 2021, while the number of patients under 40 years grew, declined by 4% in 2022, and remained the same in 2023. Thus, the pandemic affected stage IV breast cancer incidence throughout its development in both groups, although its dynamics were distinct.

This retrospective investigation found that the COVID-19 pandemic affected incidence rates, which varied by patient age. The detection rate of early cancer stages decreased, and late stages, notably stage IV, increased. These findings can be utilized to link the COVID-19 pandemic to breast cancer diagnosis delays.

## 4. DISCUSSION

According to the results above, the decline in breast cancer detection has impacted the stage at which the disease is identified in both patients under and over 40 years of age. In both age groups, there has been a noticeable shift with fewer cases being detected at early stages and an increase in the identification of later-stage cases, particularly stage IV. Similar conclusions were reached by Resende *et al*,^19^ who studied data of 11,753 patients from 2018 to 2021 and recorded a significant increase in the number of cases of late-stage breast cancer. The trend towards an increase in the detection of late-stage breast cancer was also identified by Li *et al*.^20^ It is possible to agree with the results of the scientists, but it is worth noting that their systematic review included data from 2020 to 2021, so it assessed the impact of the pandemic on the stage of cancer detection only, without covering the further dynamics of its development.

Given the pandemic’s impact on breast cancer epidemiology, it’s important to examine its causes and effects.^21^ Most researchers attribute the pandemic’s impact to the decline in preventative cancer diagnostics, particularly breast cancer screenings.^23,23^ The impact of COVID-19 on the number of regular cancer screenings was studied by Lee et al.^24^ They found that preventive diagnostic measures for stomach, colorectal, breast, and cervical cancers had significantly reduced from 2019 to 2020. The breast cancer decline was 12%. Large city dwellers, those with higher socioeconomic levels, and non-chronically ill people had much lower indicators of this parameter. While agreeing with the authors’ findings, demographic, social, and clinical variables should be analyzed to identify risk groups for decreased breast cancer screening rates.

Mendes *et al* ^25^ determined that the decrease in the number of preventive diagnostic measures to detect breast cancer during the first two years of the pandemic was 13%. The application of the prognostic model showed that the consequences of such a decline could include 506 additional deaths, 11 months of patient life-shortening, and 12,800 lost life years for the national population over the next 25 years. The results are supported by the author’s conclusions about the importance of predicting the consequences of reducing women’s preventive screening programs due to the impact of the pandemic, as they encourage the adoption of preventive measures and the resumption of routine diagnostic procedures to reduce breast cancer rates.

After analyzing the national mammography database of the United States, Grimm *et al* ^26^ assessed the impact of the pandemic on breast imaging. The study found that during the pandemic, preventive mammograms were 36.3% of pre-pandemic procedures, diagnostic mammograms were 57.9%, biopsies were 47.3%, and cancer diagnostic procedures were 48.7%. The number of breast cancer preventive diagnostic measures has increased marginally since the pandemic, but it has not reached pre-pandemic levels. Based on this study, reducing routine and diagnostic procedures for breast cancer detection is the main factor in reducing the number of detected cases, which, due to the cancer’s complexity, worsens the prognosis.

da Cunha and Antunes^27^ faced difficulties in determining the impact of the pandemic on cancer mortality. According to their study, from 2020 to 2022, cancer mortality was lower than projected. Coronavirus kills many cancer patients, according to scientists. The forecast did not account for the direct impact of the COVID-19 pandemic on cancer patients’ SARS-CoV-2 virus infection, which may have caused the discrepancy between expected and actual cancer mortality rates. The consequences of the direct impact of the pandemic were studied by Alagoz *et al* .^28^ They analyzed how COVID-19 can affect the delay of cancer surgery. According to the researchers, the likelihood of delayed surgery was higher in the group of patients diagnosed with cancer in 2020, as they had to be tested for COVID-19 and, if confirmed, treated for coronavirus.

The COVID-19 epidemic has strained world healthcare. Doctors, scientists, and other healthcare professionals have worked to curb the spread of SARS-CoV-2 and reduce its public health impact.^29^ After the pandemic, it is vital to analyze the causes and effects of COVID-19 at the global and local levels and its impact on other life-threatening diseases.^30^ Understanding the reasons and extent of this impact can help build crucial healthcare sector strategies, including clinical task prioritization. Extraneous factors may skew the results of this study, making it difficult to isolate the pandemic’s impact on cancer incidence and detection rates.

This study was limited to a single oncology center, which may not fully represent regional or national variations in healthcare access, cancer detection practices, or pandemic-related disruptions. Additionally, the retrospective design relied on existing medical records, which may be subject to incomplete data or reporting bias. Future research should involve multicenter studies with broader geographic coverage and include variables such as screening program participation, healthcare accessibility, and socioeconomic factors to more comprehensively assess the pandemic’s impact on breast cancer incidence and staging.

## 5. CONCLUSION

The COVID-19 pandemic did not affect stage 0 breast cancer detection in patients under 40 years. Stage I cancer cases declined in 2020 and started to rise in 2021 before declining linearly until the pandemic ended. Since 2020, stage IIa has declined, and stage IIb has expanded. Throughout the pandemic, stage IIIa cancer cases declined, but stage IIIb cases only fell at the start. Stage IIIc instances surged early in the pandemic. The greatest impact from 2020 to 2024 was an increase in stage IV breast cancer identification. The pandemic did not affect stage 0 cancer diagnosis in patients over 40 years, but it did reduce stage I, stage IIa, and stage IIb in the first year. Stage IIIa cases increased at the start of the pandemic, stage IIIb cases increased from 2021 to 2023, and stage IV cases increased significantly.

## ETHICS APPROVAL

The study was conducted in compliance with all ethical standards stipulated by the Declaration of Helsinki and the internal documents of the institution.

## AUTHORS’ CONTRIBUTION

AZ: Idea/Concept; AZ: Control/Supervision; AZ, IR, KM: Data Collection and/or Processing; AZ, KM: Analysis and/or Interpretation; AZ, KM: Literature Review; AZ, IR: Writing the Manuscript; IR, KM: Critical Review; KM, IR: Materials.

## INFORMED CONSENT STATEMENT

Since the study did not involve direct interaction with patients, no formal consent was required to use their medical data.

## CONFLICT OF INTEREST

The authors have no competing interests to declare.

## Figures and Tables

**Table 1.** Breast cancer incidence at Bydgoszcz Oncology Centre: age groups, clinical stages, and metastasis status before and after 2020.

Total	Incidence of breast cancer				*p* value
	<2020		≥2020		
	(2016–2019)		(2020–2023)		
	*n* (%)		*n* (%)		
	3,476	46,15	4,056	53,85	
**Age group**					
≤39 years old	165	(4.74%)	232	(5.72%)	<0.001
40–49 years old	533	(15.33%)	755	(18.61%)	
50–59 years old	935	(26.90%)	967	(23.84%)	
60–69 years old	1,258	(36.19%)	1,315	(32.42%)	
70–79 years old	454	(13.06%)	652	(16.07%)	
≥80 years old	131	(3.77%)	135	(3.33%)	
40-year-old group					
≤40 years old	195	(5.61%)	278	(6.85%)	0.027
>40 years old	3,281	(94.39%)	3,778	(93.15%)	
**Clinical stage**					
0	57	(1.64%)	28	(0.69%)	<0.001
I	1,150	(33.08%)	1,261	(31.09%)	
IIA	840	(24.17%)	987	(24.33%)	
IIB	463	(13.32%)	564	(13.91%)	
IIIA	223	(6.42%)	271	(6.68%)	
IIIB	267	(7.68%)	309	(7.62%)	
IIIC	116	(3.34%)	118	(2.91%)	
IV	360	(10.36%)	518	(12.77%)	
**N0 vs. N(+)**					
N0	2,104	(60.53%)	2,432	(59.96%)	0.615
N(+)	1,372	(39.47%)	1,624	(40.04%)	
**M0 vs. M1**					
M0	3,116	(89.64%)	3,538	(87.23%)	0.001
M1	360	(10.36%)	518	(12.77%)	

Note: 0–IV: Stage of breast cancer; N0: No cancer cells found in nearby lymph nodes; N(+): Cancer cells present in nearby lymph nodes (node-positive); M0: No distant metastasis detected (cancer has not spread to distant parts of the body); M1: Distant metastasis present (cancer has spread to distant organs or tissues). *P*-values represent the statistical significance of differences between the two time periods for each characteristic (age groups, 40-year-old groups, clinical stages, nodal status, and metastasis status).

**Table 2.** Breast cancer incidence at Bydgoszcz Oncology Centre: comparing clinical stages and metastasis status before and after 2020 in patients =40 and >40 years.

Total	Incidence of breast cancer					*p*-value
	<2020		≥2020						
	(2016–2019)		(2020–2023)		
	n (%)		n (%)		
	195	(41.23%)	278	(58.77%)	
**Clinical stage** ≤40 years old						
	0	2	(1.03%)	3	(1.08%)	0.957
	I	37	(18.97%)	57	(20.50%)	
	IIA	57	(29.23%)	71	(25.54%)	
	IIB	44	(22.56%)	66	(23.74%)	
	IIIA	15	(7.69%)	16	(5.76%)	
	IIIB	12	(6.15%)	18	(6.47%)	
	IIIC	5	(2.56%)	10	(3.60%)	
	IV	23	(11.79%)	37	(13.31%)	
**N0 vs. N(+)**						
	N0	101	(51.79%)	143	(51.44%)	0.939
	N(+)	94	(48.21%)	135	(48.56%)	
**M0 vs. M1**						
	M0	172	(88.21%)	241	(86.69%)	0.626
	M1	23	(11.79%)	37	(13.31%)	
	Total	3,281	(46.48%)	3,778	(53.52%)	
**Clinical stage** >40 years old
	0	55	(1.68%)	25	(0.66%)	<0.001
	I	1,113	(33.92%)	1,204	(31.87%)	
	IIA	783	(23.86%)	916	(24.25%)	
	IIB	419	(12.77%)	498	(13.18%)	
	IIIA	208	(6.34%)	255	(6.75%)	
	IIIB	255	(7.77%)	291	(7.70%)	
	IIIC	111	(3.38%)	108	(2.86%)	
	IV	337	(10.27%)	481	(12.73%)	
**N0 vs. N(+)**						
	N0	2,003	(61.05%)	2,289	(60.58%)	0.692
	N(+)	1,278	(38.95%)	1,489	(39.41%)	
**M0 vs. M1**						
	M0	2,944	(89.73%)	3,297	(87.27%)	0.001
	M1	337	(10.27%)	481	(12.73%)	

## References

[bib1] Guthmuller S, Carrieri V, Wübker A (2023). Effects of organized screening programs on breast cancer screening, incidence, and mortality in Europe. J Health Econ.

[bib2] Lofters AK, Wu F, Frymire E, Kiran T, Vahabi M, Green ME (2023). Cancer screening disparities before and after the COVID-19 pandemic. JAMA Netw Open.

[bib3] Star J, Bandi P, Siegel RL, Han X, Minihan A, Smith RA (2023). Cancer screening in the United States during the second year of the COVID-19 pandemic. J Clin Oncol.

[bib4] Fedewa SA, Star J, Bandi P, Minihan A, Han X, Yabroff KR (2022). Changes in cancer screening in the US during the COVID-19 pandemic. JAMA Netw Open.

[bib5] Eijkelboom AH, de Munck L, Larsen M, Bijlsma MJ, Tjan-Heijnen VCG, van Gils CH (2023). Impact of the COVID-19 pandemic on breast cancer incidence and tumor stage in the Netherlands and Norway: a population-based study. Cancer Epidemiol.

[bib6] Nguyen DL, Ambinder EB, Myers KS, Oluyemi E (2022). Addressing disparities related to access of multimodality breast imaging services before and during the COVID-19 pandemic. Acad Radiol.

[bib7] Cairns A, Jones VM, Cronin K, Yocobozzi M, Howard C, Lesko N (2022). Impact of the COVID-19 pandemic on breast cancer screening and operative treatment. Am Surg.

[bib8] Negrao EMS, Cabello C, Conz L, Mauad EC, Zeferino LC, Vale DB (2022). The COVID-19 pandemic impact on breast cancer diagnosis: a retrospective study. Rev Bras Ginecol Obstet.

[bib9] Voigtländer S, Hakimhashemi A, Grundmann N, Radespiel-Tröger M, Inwald EC, Ortmann O (2023). Impact of the COVID-19 pandemic on reported cancer diagnoses in Bavaria, Germany. J Cancer Res Clin Oncol.

[bib10] Guével E, Priou S, Lamé G, Wassermann J, Bey R, Uzan C (2023). Impact of the COVID-19 pandemic on clinical presentation, treatments, and outcomes of new breast cancer patients: a retrospective multicenter cohort study. Cancer Med.

[bib11] Eijkelboom AH, de Munck L, Lobbes MBI, van Gils CH, Wesseling J, Westenend PJ (2021). Impact of the suspension and restart of the Dutch breast cancer screening program on breast cancer incidence and stage during the COVID-19 pandemic. Prev Med.

[bib12] General Assembly of the World Medical Association (2014). World Medical Association Declaration of Helsinki: ethical principles for medical research involving human subjects. J Am Coll Dent.

[bib13] Romaszko-Wojtowicz A, Tokarczyk-Malesa K, Doboszyńska A, Glińska-Lewczuk K (2024). Impact of COVID-19 on antibiotic usage in primary care: a retrospective analysis. Sci Rep.

[bib14] Lombardi A, Vitale V, Nigri G, Olivieri C, Mastrangeli MR, Bizzaglia E (2020). Prognostic impact of the 8th edition of American Joint Committee on Cancer (AJCC) cancer staging system on clinically negative lymph nodes (cN0) breast cancer patients. Breast J.

[bib15] Didkowska J, Wojciechowska U, Michalek IM, Caetano dos Santos FL (2022). Cancer incidence and mortality in Poland in 2019. Sci Rep.

[bib16] DeSantis CE, Ma J, Gaudet MM, Newman LA, Miller KD, Sauer AG (2019). Breast cancer statistics, 2019. CA Cancer J Clin.

[bib17] Ferlay J, Partensky C, Bray F (2016). More deaths from pancreatic cancer than breast cancer in the EU by 2017. Acta Oncol.

[bib18] Gawrych M, Cichoń E, Kiejna A (2021). COVID-19 pandemic fear, life satisfaction and mental health at the initial stage of the pandemic in the largest cities in Poland. Psychol Health Med.

[bib19] Resende CAA, Fernandes Cruz HM, Costa E Silva M, Paes RD, Dienstmann R, Barrios CHE (2022). Impact of the COVID-19 pandemic on cancer staging: an analysis of patients with breast cancer from a community practice in Brazil. JCO Glob Oncol.

[bib20] Li T, Nickel B, Ngo P, McFadden K, Brennan M, Marinovich ML (2023). A systematic review of the impact of the COVID-19 pandemic on breast cancer screening and diagnosis. Breast.

[bib21] Lucas E, Murillo R, Arrossi S, Bárcena M, Chami Y, Nessa A (2023). Quantification of impact of COVID-19 pandemic on cancer screening programmes – a case study from Argentina, Bangladesh, Colombia, Morocco, Sri Lanka, and Thailand. eLife.

[bib22] Chiu HM, Su CW, Hsu WF, Jen GHH, Hsu CY, Chen SLS (2021). Mitigating the impact of COVID-19 on colorectal cancer screening: organized service screening perspectives from the Asia-Pacific region. Prev Med.

[bib23] Koczkodaj P, Kamiński MF, Ciuba A, Didkowska J (2021). Cancer screening coverage in Poland – From bad to better to the worst during the SARS-CoV-2 pandemic. Arch Med Sci.

[bib24] Lee K, Lee YY, Suh M, Jun JK, Park B, Kim Y (2022). Impact of COVID-19 on cancer screening in South Korea. Sci Rep.

[bib25] Mendes D, Figueiredo D, Alves C, Penedones A, Costa B, Marques FB (2023). P10 impact of the COVID-19 pandemic on cancer screenings in Portugal and years of life lost due to delayed diagnoses. Value Health.

[bib26] Grimm LJ, Lee C, Rosenberg RD, Burleson J, Simanowith M, Fruscello T (2022). Impact of the COVID-19 pandemic on breast imaging: an analysis of the national mammography database. J Am Coll Radiol.

[bib27] da Cunha AR, Antunes JLF (2024). Impact of the COVID-19 pandemic on cancer mortality in Brazil. BMC Cancer.

[bib28] Alagoz O, Lowry KP, Kurian AW, Mandelblatt JS, Ergun MA, Huang H (2021). Impact of the COVID-19 pandemic on breast cancer mortality in the US: estimates from collaborative simulation modeling. J Natl Cancer Inst.

[bib29] Chung SH, Romatoski KS, Rasic G, Beaulieu-Jones BR, Kelly Kenzik K, Merrill AL (2023). Impact of the COVID-19 pandemic on delays to breast cancer surgery: ripples or waves?. Ann Surg Oncol.

[bib30] Trifanescu OG, Gales L, Bacinschi X, Serbanescu L, Georgescu M, Sandu A (2022). Impact of the COVID-19 pandemic on treatment and oncologic outcomes for cancer patients in Romania. In Vivo.

